# Therapeutically targeting mitochondrial redox signalling alleviates endothelial dysfunction in preeclampsia

**DOI:** 10.1038/srep32683

**Published:** 2016-09-08

**Authors:** Cathal McCarthy, Louise C. Kenny

**Affiliations:** 1 The Irish Centre for Fetal and Neonatal Translational Research (INFANT), Department of Obstetrics and Gynaecology, Cork University Hospital, Wilton, Cork, Ireland

## Abstract

Aberrant placentation generating placental oxidative stress is proposed to play a critical role in the pathophysiology of preeclampsia. Unfortunately, therapeutic trials of antioxidants have been uniformly disappointing. There is provisional evidence implicating mitochondrial dysfunction as a source of oxidative stress in preeclampsia. Here we provide evidence that mitochondrial reactive oxygen species mediates endothelial dysfunction and establish that directly targeting mitochondrial scavenging may provide a protective role. Human umbilical vein endothelial cells exposed to 3% plasma from women with pregnancies complicated by preeclampsia resulted in a significant decrease in mitochondrial function with a subsequent significant increase in mitochondrial superoxide generation compared to cells exposed to plasma from women with uncomplicated pregnancies. Real-time PCR analysis showed increased expression of inflammatory markers TNF-α, TLR-9 and ICAM-1 respectively in endothelial cells treated with preeclampsia plasma. MitoTempo is a mitochondrial-targeted antioxidant, pre-treatment of cells with MitoTempo protected against hydrogen peroxide-induced cell death. Furthermore MitoTempo significantly reduced mitochondrial superoxide production in cells exposed to preeclampsia plasma by normalising mitochondrial metabolism. MitoTempo significantly altered the inflammatory profile of plasma treated cells. These novel data support a functional role for mitochondrial redox signaling in modulating the pathogenesis of preeclampsia and identifies mitochondrial-targeted antioxidants as potential therapeutic candidates.

Preeclampsia is a pregnancy-specific syndrome that complicates 5% of nulliparous pregnancies and worldwide affects approximately 4 million women per annum^1^. Globally, preeclampsia is a leading cause of maternal mortality and it is responsible for occupancy of approximately 20% of neonatal intensive care unit cots. The syndrome is characterized clinically by maternal hypertension accompanied by proteinuria or haematological or biochemical abnormalities^1^. Despite intense research, the precise pathophysiological mechanisms underlying this syndrome remain poorly elucidated.

However, there is substantial evidence that defective placentation in early pregnancy is a pivotal event in the aetiology of this condition^2^. A consequential reduction in placental perfusion provokes an ischemic placental microenvironment due to fluctuations in oxygen delivery to the placenta and fetus, which results in oxidative stress^3^. Elevated placental oxidative stress is evident in preeclampsia as early as ~8–10 weeks’ gestation^4^. Placental ischemia is inherently linked to elevated production and secretion of placental-derived deleterious mediators that induce widespread maternal endothelial dysfunction. Uncomplicated pregnancy is itself a state of oxidative stress^5^ as a result of amplified maternal metabolism and the subsequent metabolic activity of the placenta^6^. However, during preeclampsia the mitigative systems normalizing the placental oxidative state are distorted, leading to elevated generation of pathogenic factors and subsequent vascular dysfunction. During preeclampsia, oxidative stress manifests in both the placenta and maternal circulation^7^, with evidence of diminished antioxidant defences^8^, increased free radical formation and isoprostanes^9^.

There are several sources of reactive oxygen species (ROS) within the cell; however mitochondria are the dominant cellular producers of ROS. Recent evidence has established that mitochondrial-ROS (mROS) have evolved as raconteurs directing mitochondrial function and other critical physiological signalling roles to preserve homeostasis and stimulate adaption to deleterious stressors^10^. There is a growing body of evidence implicating mitochondrial dysfunction as a pathogenic mediator of oxidative stress in preeclampsia^11^. There is substantial mitochondrial content in the placenta, in part to mediate the elevated metabolic activities during pregnancy^12^. Excessive production of mitochondrial-ROS is intrinsically linked to mitochondrial dysfunction^13^,^14^. Furthermore, there is a higher incidence of preeclampsia in a family with pre-diagnosed mitochondrial dysfunction^15^.

MitoTempo is a mitochondria-targeted superoxide dismutase antioxidant mimetic. Recent work demonstrated that MitoTempo accumulates in the mitochondria by increasing mitochondrial O_2_^−^dismutation while not affecting cytoplasmic dismutation in endothelial cells^16^. Additionally, MitoTempo decreased mitochondrial O_2_^−^in intact endothelial cells. Furthermore, MitoTempo improved endothelial function and reduced mROS production in an *in vivo* model of hypertension^16^. In the current studies, we identified that deleterious plasma mediators present in preeclampsia generate increased mitochondrial-specific superoxide production, by treating HUVEC with pooled plasma from preeclampsia pregnancies and matched uncomplicated controls from the SCOPE study (www.scopestudy.net) and non-pregnant controls. We show that increased mROS production evoked vascular dysfunction. Finally, we determine that antioxidants (MitoTempo) directly targeting mitochondrial superoxide scavenging prevent increased mROS production and elucidate the potential novel therapeutic pathway that may treat the syndrome more effectively.

## Results

### Preeclampsia plasma mediators modulate mitochondrial metabolism in HUVEC

**Figure 1:**
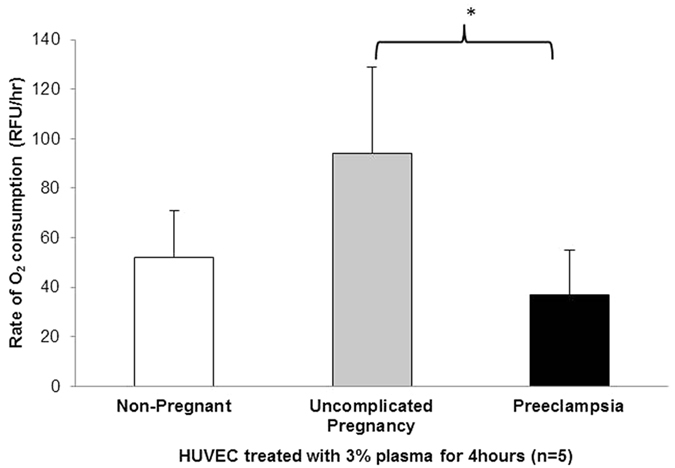
Preeclampsia plasma alters mitochondrial metabolism in plasma treated HUVEC. (**a**) Human umbilical vein endothelial cells were incubated with 3% plasma from women with preeclampsia, uncomplicated pregnancies and non-pregnant women for 4 hrs. Oxygen consumption rate was measured with MitoXpress fluorogenic probe. Time-resolved fluorescence of each well was then measured every 2 minutes for a total of 180 minutes. Rate of oxygen consumption was determined from the slope of fluorescence vs. time for each sample using relative fluorescence units/hour. Data are expressed as mean ± SEM. (*P < 0.05 vs normal pregnancy). Data are representative of 5 independent experiments.

The emerging role of mitochondrial dysfunction in mediating the pathogenesis of preeclampsia, led us to investigate a potential link between deleterious plasma mediators in preeclampsia and a subsequent dysregulation of mitochondrial function in HUVEC. In our first set of experiments we examined mitochondrial respiration by measuring oxygen consumption in plasma-treated HUVEC using the MitoXpress assay containing an oxygen sensitive fluorescent probe. Rates of oxygen consumption (OCR) are calculated from the changes in fluorescence signal over time using fluorescence plate reader. We established that after 4 hrs incubation of HUVEC with 3% plasma from women with preeclampsia significantly reduced OCR (40.61 ± 18.10 RFU, n = 5, P < 0.05) when compared with treatment with 3% plasma from uncomplicated pregnant (94.12 ± 34.9 RFU, n = 5) and non-pregnant women (52.12 ± 18.8, n = 5) (Fig. 1).

### Preeclampsia plasma mediators alter PGC-1α expression in HUVEC

**Figure 2:**
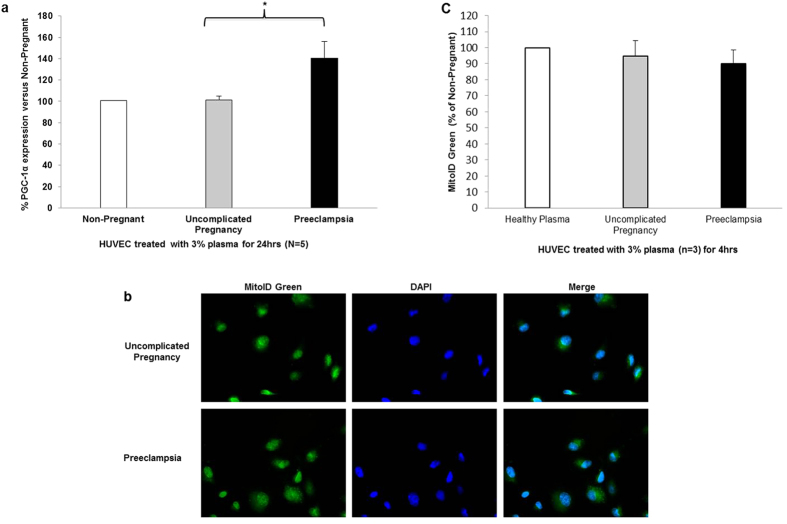
Determination of mitochondrial biogenesis/mass in plasma treated HUVEC. (**a**) PGC-1α protein expression was detected by fluorescent microscopy and quantified using Image J software. Data is the mean of 5 independent experiments and are expressed as difference in percentage pixel intensity between the study groups ± SEM. *P < 0.05. (**b**) Mitochondrial mass was determined using fluorogenic MitoID Green reagent. Confocal microscopy of MitoID Green (green fluorescence, 1st panel) and Hoechst 33342 (blue fluorescence, 2nd panel) at 20X. Merged image localizes mitochondria (3rd panel). (**c**) Mitochondrial mass (MitoID Green intensity) was quantified using Image J software. Data is the mean of 3 independent experiments and are expressed as difference in percentage pixel intensity between the study groups.

Given that peroxisome proliferator activated receptor γ co-activator 1-α (PGC-1α) mediates mitochondrial biogenesis and antioxidant activity in HUVEC; we determined the effect of preeclampsia plasma on protein expression of PGC-1α in HUVEC. Preeclampsia plasma stimulated a significant increase in PGC-1α protein expression (140.63% ± 13.5%, n = 5, P < 0.05) when compared with treatment with 3% plasma from uncomplicated pregnant (101.08% ± 3.97%, n = 5) and non-pregnant women (100% ± 0%, n = 5) (Fig. 2a). Additionally, we measured the effect of preeclampsia plasma on mitochondrial mass in treated HUVEC using MitoID Green by fluorescent microscopy. This fluorogenic reagent preferentially accumulates in mitochondria becoming fluorescent in their lipid environment. There was no significant difference in mitochondrial mass (89% ± 8.63, n = 3) when compared with treatment with 3% plasma from uncomplicated pregnant (94.91% ± 9.46%, n = 3) and non-pregnant women (100% ± 0%, n = 3) (Fig. 2b,c).

### Preeclampsia plasma increases mitochondrial-specific ROS generation in HUVEC

**Figure 3:**
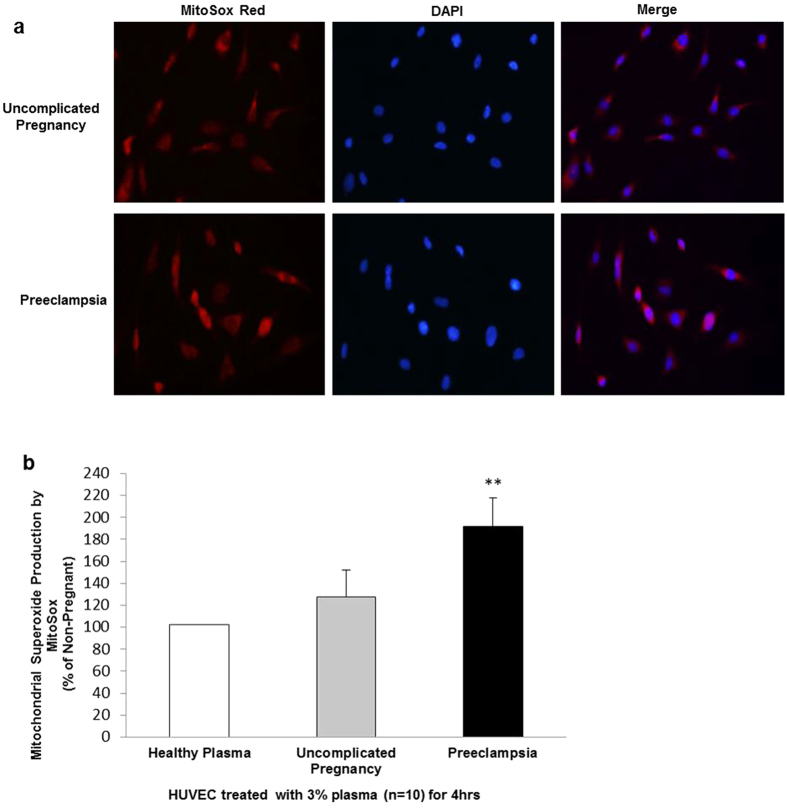
Detection of mitochondrial-specific superoxide in plasma treated HUVEC. HUVEC were incubated with 3% plasma from women with preeclampsia, uncomplicated pregnancies and non-pregnant women for 4 hrs and mitochondrial-specific superoxide was detected using fluorogenic MitoSox Red dye. (**a**) Confocal microscopy of MitoSox (red fluorescence, 1st panel) and DAPI (blue fluorescence, 2nd panel) at 20X. Merged image localizes mitochondrial superoxide production (3rd panel). (**b**) MitoSox Red generation was quantified using Image J software. Data is the mean of 10 independent experiments and are expressed as difference in percentage pixel intensity between the study groups ± SEM. **P < 0.01.

Excessive production of mitochondrial-ROS is intrinsically linked to mitochondrial dysfunction. To measure mitochondrial-specific superoxide production, plasma-treated cells were labelled with MitoSox Red dye and measured by fluorescent microscopy. Therefore, in our next set of experiments (Fig. 3a,b), we observed that treatment of HUVEC with 3% plasma from women with preeclampsia significantly increased mitochondrial-specific superoxide generation (191.82% ± 25%, n = 10, P <  0.05) when compared with treatment with 3% plasma from uncomplicated pregnant (127.65% ± 24%, n = 10) and non-pregnant women (100% ± 0%, n = 10) (Fig. 3b).

### Effect of preeclampsia-plasma mediators on endogenous endothelial antioxidant and inflammatory markers

**Figure 4:**
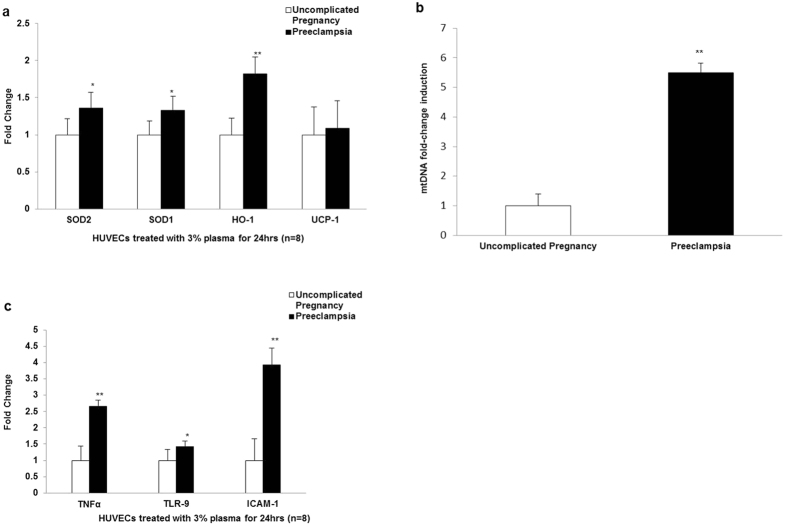
Preeclampsia plasma alters endothelial antioxidant and inflammatory profiles. HUVEC were incubated with 3% plasma from women with preeclampsia and uncomplicated pregnancies for 24 hrs and gene expression of antioxidant and inflammatory markers were quantified by real-time PCR. (**a**) Gene expression of redox signalling mediators, SOD1, SOD2, HO-1 and UCP-1 respectively was determined in plasma-treated HUVEC. The amounts of amplified products are expressed relative to geometric mean of two internal controls. Data are representative of 8 independent experiments. (**b**) Real-time PCR quantifying the levels of mtDNA relative to nuclear DNA in plasma from preeclampsia and uncomplicated pregnancies respectively. Data are mean fold change compared to uncomplicated pregnancy ± SEM. **P < 0.01. Data are representative of 12 independent experiments. (**c**) Gene expression of inflammatory markers TNF-α, TLR-9 and ICAM-1 respectively was determined in plasma-treated HUVEC. The amounts of amplified products are expressed relative to geometric mean of two internal controls. Data are representative of 8 independent experiments. Data are mean fold change compared to uncomplicated pregnancy ± SEM. *P < 0.05; **P < 0.01.

Given the proficiency of mROS in regulating various signalling pathways and consequent pathologies, the effect of plasma mediators on antioxidant gene expression was determined by quantitative real-time PCR in HUVEC. Incubation with preeclampsia plasma induced a significant increase in SOD1 gene expression (1.32 ± 0.10 fold, n = 8, P < 0.05), SOD2 gene expression (1.35 ± 0.21 fold, n = 8, P < 0.05) and HO-1 gene expression (1.82 ± 0.42 fold, n = 8, P < 0.05) respectively compared to uncomplicated pregnancy (Fig. 4a).

Mitochondrial damage associated molecular patterns (DAMPs) including mtDNA are ligands for TLR-9-induced inflammation^14^,^17^. The levels of mtDNA relative to nuclear DNA were measured in plasma from preeclampsia and uncomplicated pregnancies respectively. Preeclampsia plasma had a significant increase in mtDNA (5.49 ± 0.33 fold, n = 12, P < 0.01) compared to plasma from uncomplicated pregnancy (Fig. 4b). Subsequently, preeclampsia plasma mediators significantly increased enodthelial TLR-9 gene expression (1.43 ± 0.17 fold, n = 8, P < 0.01) and pro-inflammatory TNF-α gene expression (2.65 ± 0.17 fold, n = 8, P < 0.01) in HUVEC respectively, compared to uncomplicated pregnancy. Additionally, preeclamspia plasma mediators increased endothelial dysfunction as evident from the significant increase in gene expression of ICAM-1 (3.92 ± 0.51 fold, n = 8, P < 0.01) in HUVEC respectively, compared to uncomplicated pregnancy (Fig. 4c).

### Cytoprotective effects of MitoTempo on H_2_O_2-_induced cell death

**Figure 5:**
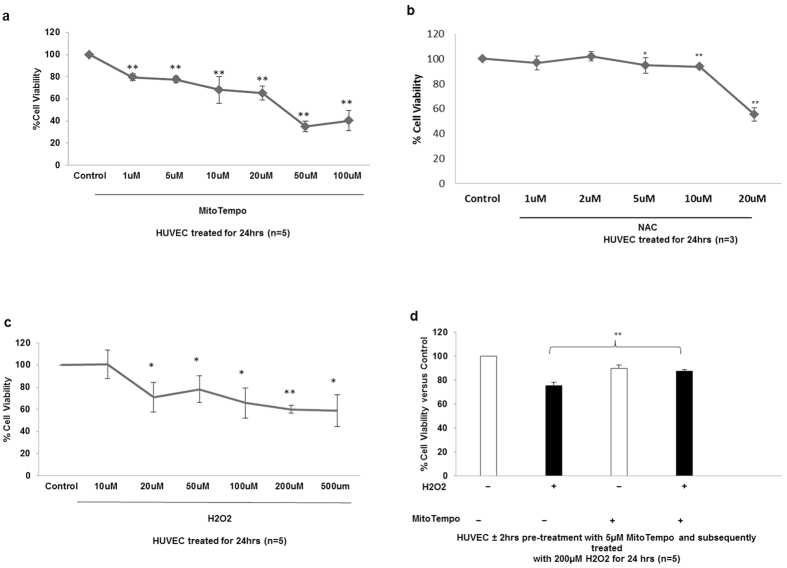
MitoTempo reduces H2O2 mediated cell death. (**a**) Dose dependent effect of MitoTempo on HUVEC cell viability was assessed using a MTT assay. HUVEC were treated with varying concentrations of MitoTempo for 24 hrs. Data are expressed as mean ± SEM. (*P < 0.05; **P < 0.01 vs control). Data are representative of 5 independent experiments. (**b**) Dose dependent effect of NAC on HUVEC cell viability was assessed using a MTT assay. HUVEC were treated with varying concentrations of NAC for 24 hrs. Data are expressed as mean ± SEM. (*P < 0.05; **P < 0.01 vs control). Data are representative of 3 independent experiments. (**c**) Dose dependent effect of H2O2 on HUVEC cell viability was assessed using an MTT assay. HUVEC were treated with increasing concentrations of H2O2 for 24 hours. Data are expressed as mean ± SEM. (*P  <  0.05; **P < 0.01 vs control). Data are representative of 5 independent experiments. (**d**) MitoTempo is protective of H2O2-induced cell death in HUVEC. Cells were pre-treated for 2 hrs with 5 μM MitoTempo and subsequently exposed to 200 μM H2O2 for 24 hrs, cell viability was assessed by MTT assay. Data are expressed as mean ± SEM. (*P < 0.05; vs control). Data are representative of 5 independent experiments.

The aim of the next series of experiments was to analyse the protective properties of mitochondrial targeted antioxidant, MitoTempo in HUVEC in response to cellular stressors. Initially we determined the optimal concentration of MitoTempo that did not adversely affect cell viability. HUVEC were treated with increasing concentrations of MitoTempo and cell viability was determined using an MTT assay. In summary, 5 uM MitoTempo was used in subsequent experiments (Fig. 5a). Additionally, using an MTT assay we determined that 5 uM of non-mitochondrial targeted N-acetylcysteine (NAC) was the optimal concentration that did not adversely affect HUVEC cell viability (Fig. 5b).

Furthermore, H_2_O_2_ is frequently used as a cellular stressor to mimic oxidative stress in *in vitro* cellular systems and is a potential pathogenic mediator present in the preeclampsia plasma milieu. To determine the optimal dose of H_2_O_2_ that reduces cell viability in HUVEC, cells were treated with increasing concentrations of H_2_O_2_ and cell viability was recorded by MTT assay. A significant reduction in cell viability was seen in cells treated with a range of 20 uM-500 uM H_2_O_2_ compared to control (*P < 0.05 and **P < 0.01). 200 uM H_2_O_2_ was chosen as the optimal dose to use in subsequent experiments as it significantly reduced HUVEC cell viability by approximately 35% (Fig. 5c).

Having determined that treatment of HUVEC with 200 uM H_2_O_2_ significantly reduced cell viability, we sought to establish if pre-treating cells with 5 uM MitoTempo could neutralize these destructive effects. Pre-treatment with 5 uM MitoTempo rescued H_2_O_2_-induced cell death (87.23% ± 1.51% vs 75.32% ± 2.68%, n = 5, P < 0.05) establishing the therapeutic capacity of mitochondrial-targeted antioxidants in reducing H_2_O_2_ cytotoxicity (Fig. 5d).

### MitoTempo reduces preeclampsia plasma mediated increase in mitochondrial-specific ROS generation in HUVEC

**Figure 6:**
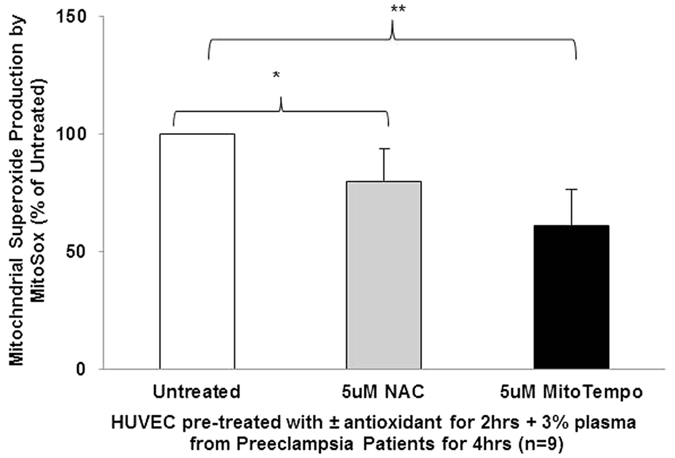
MitoTempo scavenging reduces mROS production. HUVEC were pre-treated with 5 μM MitoTempo or 5 μM NAC for 2 hrs prior to incubation with 3% plasma from women with preeclampsia for 4 hrs and mitochondrial-specific superoxide production was detected using fluorogenic MitoSox Red dye. MitoSox Red generation was quantified using Image J software. Data is the mean of 9 independent experiments and are expressed as difference in percentage pixel intensity compared to untreated ± SEM, *P < 0.05, **P < 0.01.

We examined if pre-incubation with MitoTempo could scavenge the volume of mROS production in HUVEC exposed to preeclampsia plasma mediators. To elucidate the proficiency of mitochondrial-specific antioxidants, we additionally included non-mitochondrial targeted N-acetylcysteine (NAC) in our next experiments. Cells were pre-treated with 5 μM MitoTempo or 5 μM NAC for 2 hrs prior to exposure to 3% plasma from women with preeclampsia and levels of mitochondrial superoxide were detected by fluorogenic MitoSox Red dye and analysed using Image J software. Pre-treatment with MitoTempo significantly reduced mROS generation compared to untreated cells (61.23% ± 15.42% vs 100% ± 0%, n = 9, P < 0.01). Pre-treatment with non-targeted antioxidants reduced mROS production (79.73% ± 13.97% vs 100% ± 0%, n = 9, P < 0.05) but its effects are not as potent as targeted mitochondrial antioxidants (Fig. 6).

### MitoTempo mediates redox and inflammatory signals in response to cellular stressors

**Figure 7:**
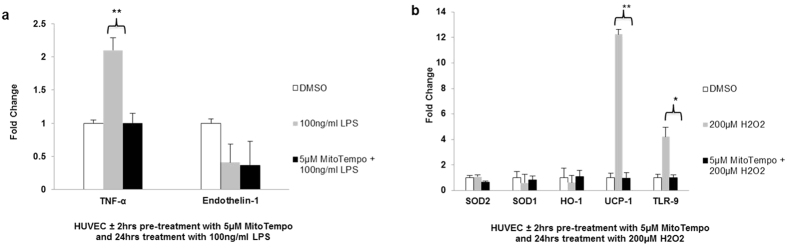
Effect of MitoTempo on inflammatory and redox signalling markers in HUVEC. HUVEC were pre-treated with 5 μM MitoTempo for 2 hrs prior to exposure to 100 ng/ml LPS for 24 hrs and gene expression of inflammatory markers were quantified by real-time PCR. (**a**) Gene expression of inflammatory markers TNF-α and endothelin-1 respectively was determined in LPS-stimulated HUVEC. Cells were pre-treated with 5 μM MitoTempo for 2 hrs prior to exposure to 200 μM H2O2 for 24hrs and gene expression of redox markers were quantified by real-time PCR. (**b**) Gene expression of redox signalling mediators, SOD1, SOD2, HO-1, UCP-1and TLR-9 respectively were determined in HUVEC following oxidative stress insult. The amounts of amplified products are expressed relative to geometric mean of two internal controls. Data are mean fold change compared to control ± SEM. *P < 0.05; **P < 0.01. Data are representative of 3 independent experiments.

To specifically elucidate the protective effects of mitochondrial targeted antioxidant, MitoTempo in regulating pathogenic cellular pathways in endothelial cells, HUVEC were pre-treated with MitoTempo prior to exposure to stressors including H_2_O_2_ (oxidative stress) and LPS (inflammation). Firstly, we examined the expression of inflammatory markers in response to 24 hrs stimulation with LPS (100 ng/ml) with/without 2 hrs MitoTempo (5 μM) pre-treatment. There was a significant decrease in TNF-α gene expression (2.09 ± 0.18 fold vs 0.99 ± 0.15 fold, n = 3, P < 0.01) (Fig. 7a) in cells pretreated with MitoTempo compared to untreated cells.

Additionally, we determined the expression of redox signalling markers in response to 24 hrs stimulation with H_2_O_2_ (200 μM) with/without 2 hrs MitoTempo (5 μM) pre-treatment. There was a significant decrease in UCP-1 gene expression (12.23 ± 0.39 fold vs 0.95 ± 0.43 fold, n = 3, P < 0.01) in cells pretreated with MitoTempo compared to untreated cells. Uncoupling proteins mediate the electrochemical potential across the inner mitochondrial membrane, which can subsequently regulate mROS production. Furthermore, there was a significant decrease in TLR-9 gene expression (4.2 ± 0.73 fold vs 1.02 ± 0.19 fold, n = 3, P < 0.05), demonstrating a novel role for MitoTempo in reducing mROS-mediated innate immune response (Fig. 7b).

## Discussion

In excess of 800 peer-reviewed publications over the past 25 years have corroborated the hypothesis that oxidative damage is involved in the pathophysiology of preeclampsia yet current antioxidant interventions are not clinically effective. One possible explanation is that these antioxidant regimens have failed to reach the intracellular location, namely the mitochondria; hence they have failed to ameliorate the pathological oxidative damage. Mitochondrial pharmacology has recently greatly advanced with a number of different pharmacology strategies in development to address mitochondrial dysfunction.

Mitochondrial dysfunction is a pathogenic mediator of oxidative stress in preeclampsia with increased mitochondrial lipid peroxidation and enhanced susceptibility to oxidation evident in mitochondria in the placenta of pregnancies complicated by preeclampsia^18^. Endothelial cells are the primary targets of the circulating factors and preeclampsia is characterised by aberrant vascular dysfunction^19^. There are a number of different instigators that distort mitochondrial function, including altered oxygen consumption, decreased ATP production, increased mROS production and mtDNA damage^20^. We explored the pathogenic mechanisms of preeclampsia plasma mediators on mitochondrial function by assessing oxygen consumption rates. We showed that preeclampsia plasma mediators significantly reduced mitochondrial respiration compared to uncomplicated pregnancy. This corroborates with recent work that showed alterations in mitochondrial morphology in preeclampsia and highlighted that miR210 (increased in preeclampsia) is potentially responsible for repression of mitochondrial respiration in preeclampsia^21^.

In the present study we show that mitochondrial-ROS is markedly elevated in HUVEC treated with plasma from pregnancies complicated by preeclampsia compared to uncomplicated pregnancies. Increased mROS production may be caused by decreased cellular respiration observed in OCR assays as impaired cellular respiration can lead to backward flux of electrons in the oxidative phosphorylation chain^22^. Likewise analysis of the mitochondrial placental proteome in preeclampsia reported increased abundance of proteins involved in oxidative stress and ROS generation^23^. Furthermore, recent work in a transgenic murine model overexpressing storkhead box 1 (preeclampsia susceptibility gene) showed exaggerated placental mitochondrial activity^24^.

PGC-1α is a well characterised pleiotropic orchestrator of mitochondrial biogenesis and antioxidant activity^25^. We showed increased PGC-1α protein expression in HUVEC treated with plasma form preeclampsia pregnancies. The dynamics of mitochondrial biogenesis and function is a complex system of cellular and molecular processes. Mitochondrial mass signifies the equilibrium between rates of biogenesis and degradation^26^. We measured mitochondrial mass and found no significant difference in mitochondrial mass in HUVEC treated with preeclampsia plasma when compared with plasma from uncomplicated pregnancy. The lack of a change in mitochondrial mass could be related to increased rate of degradation; interestingly recent data has shown increased autophagy in preeclampsia and in HUVEC’s exposed to oxidative stress^27^. These results would suggest that preeclampsia plasma mediators alter mitochondrial metabolism and provoke mitochondrial dysfunction through multiple mechanisms.

Mitochondrial-ROS production is stringently regulated by numerous antioxidant systems in order to maintain redox-signalling homeostasis. To determine if these antioxidant pathways were modulated in HUVEC treated with plasma from preeclampsia pregnancies, we analysed the expression of both mitochondrial and non-mitochondrial antioxidants in the endothelium. Preeclampsia plasma significantly increased mitochondrial SOD1, SOD2 and non-mitochondrial HO-1 gene expression respectively. Previous studies have shown elevated decidual HO-1 protein expression and increased HO-1 in maternal serum from preeclampsia pregnancies^28^. Previous work has reported reduced SOD1 mRNA and SOD activity in isolated trophoblasts from preeclampsia patients^29^.

Recent reports have demonstrated that mitochondrial DAMPs (mROS and mtDNA) act as ligands for TLR-9^14^. Activation of TLR-9 signaling via mtDNA induces a subsequent inflammatory response with the synthesis of pro-inflammatory cytokines including TNF-α^14^. We showed increased levels of mtDNA in preeclampsia plasma. We also reported a significant increase in endothelial TLR-9 gene expression with a consequent increase in pro-inflammatory cytokine TNF-α gene expression respectively in HUVEC treated with preeclampsia plasma. TLR-9 expression has been shown to be significantly increased in the placenta in patients with preeclampsia^30^. Furthermore we showed a significant increase in ICAM-1 gene expression (marker of vascular dysfunction) in HUVEC treated with preeclampsia plasma. This correlates with previous work, which reported elevated ICAM-1 expression in preeclampsia^31^ and in HUVEC’s exposed to pathogenic necrotic trophoblast debris^32^. Furthermore, a recent transcriptomic study in HUVEC exposed to preeclampsia plasma established perturbation in pathways mediating endothelial homeostasis^33^. These findings implicate cross-talk between cellular stressors present in the maternal plasma milieu in preeclampsia.

MitoTempo is a mitochondria-targeted superoxide dismutase antioxidant mimetic. We observed that 5 μM MitoTempo rescued cell viability following exposure to oxidative stress (200 μM H_2_O_2_). Importantly we showed that MitoTempo significantly reduced mROS generation following exposure to plasma from preeclampsia pregnancies. Intriguingly, mitochondrial-targeted antioxidant pre-treatment was more effective than general antioxidant (NAC) at similar concentrations, highlighting the importance of a direct-targeted therapeutic approach.

In order to specifically characterise the potential signalling pathways mediated by MitoTempo, HUVEC were pre-treated with the MitoTempo prior to exposure to two recognised cell stressors (oxidative stress and inflammation) hypothesised to be present as pathogenic mediators in the preeclampsia plasma milieu. We identified that MitoTempo exerted an anti-inflammatory response following LPS stimulation with a significant reduction in TNF-α gene expression. Interestingly, MitoTempo significantly reduced TLR-9 gene expression only in response to H_2_O_2,_ implicating mROS in provoking inflammatory response in plasma-treated HUVEC. MitoTempo has previously been shown to reduce expression of pro-inflammatory cytokines in *in vivo* models of hypertension^34^. Furthermore MitoTempo significantly normalised UCP-1 gene expression. Uncoupling reduces ROS generation by decreasing the electrochemical potential across the inner mitochondrial membrane, reducing the half-life of the most reactive steps in the electron transport chain^35^. Hence our results have identified potential mechanistic pathways for mitochondrial scavengers in mediating oxidative damage.

Here we provide evidence for the first time that plasma mediators of preeclampsia dysregulate mitochondrial function, generate increased production of deleterious mROS in the endothelium and ultimately provoke inflammatory-induced vascular dysfunction. We describe a mechanism for mediating the aberrant production of these pathogenic regulators using mitochondrial-targeted antioxidants that directly scavenge mitochondrial superoxide production. Our findings delineate that these mitochondrial antioxidants facilitate this outcome by adapting mitochondrial metabolism. Thus, our study shows that MitoTempo restrains production of mROS-mediated deleterious inflammatory cellular signaling pathways and provides evidence that therapeutic strategies directly targeting mitochondrial superoxide scavenging should be actively pursued in future therapeutic studies of preeclampsia.

## Materials and Methods

### Study Subjects

Subjects were recruited from the Screening for Pregnancy Endpoints (SCOPE) study Ireland, an international multicentre prospective cohort study of nulliparous singleton pregnancies. Further details of this study have been published previously^36^. Preeclampsia was defined as systolic blood pressure ≥140 mm Hg and/or diastolic blood pressure ≥90 mm Hg on at least 2 occasions 4 hrs apart after 20 weeks’ gestation but before the onset of labor or postpartum, with proteinuria (24 hour urinary protein ≥300 mg, or urine dipstick protein ≥2+) or any multisystem complication of preeclampsia. Time-of-disease samples (n = 12) for preeclampsia were taken when women had these criteria to diagnose preeclampsia. Control blood samples were taken from healthy pregnant women with uncomplicated pregnancies (n = 12) in the SCOPE study and matched for age, body mass index (BMI), and gestational age and from non-pregnant women matched for BMI and maternal age (Supplementary Table S1). The SCOPE study was conducted according to the guidelines laid down in the Declaration of Helsinki, and all procedures were approved by the Clinical Research Ethics Committee of the Cork Teaching (ECM5(10)05/02/08), and all women provided written informed consent.

### Sample Collection

Plasma samples were thawed on ice, centrifuged at 3000rpm at 4 °C, and the soluble component was removed, the remainder of sample was agitated. An equal volume (20–50ul per sample) was pooled with other samples in a single Falcon tube and agitated thoroughly. Aliquots were divided into 30ul volumes for storage at −80 °C. In preliminary experiments, cell viability was determined using a range of plasma concentrations, 3% plasma concentration reduced the cytotoxic effects of plasma while maximizing relative differences between subject groups (Supplementary Figure S1). This concentration was used in the remainder of the study.

### Cell Culture

Human umbilical vein endothelial cells (HUVEC) (Lonza) were cultured in EBM-2 medium supplemented with cell Bullet kit (Lonza). HUVEC were passaged every four days and in all experiments cells at passage 4–8 were used.

### Cytotoxicity Assay

Cell viability was determined by the colorimetric MTT tetrazolium reduction assay. Cells were serum starved for 4hrs prior to incubation with either plasma from preeclampsia patients, MitoTempo (Enzo Life Sciences) or N-acetylcysteine (NAC) (Sigma-Aldrich), or Hydrogen peroxide (H_2_O_2_)(Sigma-Aldrich) or Dimethyl sulfoxide (DMSO) for 24 hrs. In additional experiments, cells were serum starved and pre-treated with 5 μM MitoTempo for 2 hrs prior to exposure to 200 μM H_2_O_2_ for 24 hrs. Following treatment, 10 ul of MTT (final concentration 5 mg/ml) was added to each well and absorbance read at 570 nm, with 630 nm as a reference. Cell viability % = absorbance of each treated cells/absorbance of control (DMSO) treated cells x100.

### Isolation of RNA and Real-time PCR analysis

HUVEC were serum starved for 4 hrs prior to treatment with either 3% plasma, 5 μM MitoTempo, 5 uM NAC, 200 μM H_2_O_2,_ 100 ng/ml LPS or DMSO control for 24 hrs. For MitoTempo antioxidant experiments, cells were serum starved for 4 hrs, pre-treated with 5 μM MitoTempo for 2 hrs and then treated with 3% plasma, 200 μM H_2_O_2,_ or 100 ng/ml LPS for 24 hrs. RNA was extracted using the RNeasy mini-kit (Qiagen). Superoxide dismutase 1 (SOD1), SOD2, uncoupling protein-1 (UCP-1), heme oxygenase-1 (HO-1), toll-like receptor 9 (TLR-9), tumour necrosis factor-α (TNF-α) and intracellular adhesion molecule-1 (ICAM-1) gene expression was quantified by Real-Time PCR using the StepONE Plus Detection system. Taqman assays (Applied BioSystems) and Sybr Green primers (Supplementary Table S2 online) were used for quantification. The amounts of target gene, normalized to geometric mean of two internal controls (18S and Tata Binding Protein) were determined using the 2^−ΔΔCT^.

### mtDNA Quantification

DNA was extracted from 200 μl of plasma from both preeclampsia (n = 12) and uncomplicated pregnancies (n = 12) respectively with QIAmp DNA mini kit (Qiagen) according to manufacturer’s instructions. Real-Time PCR was performed with 10 ng total DNA using the StepOne Plus Detection system. Mitochondrial DNA primers (hMitoF5, hMitoR5) and β2M nuclear genome primers (β2MF2, β2MR2) were used for quantitation and are provided in Supplementary Table S2 online. Relative quantification of mitochondrial DNA (mtDNA) over nuclear DNA levels were determined using the using the 2^−ΔΔCT^ method.

### Immunofluoresence

HUVEC were serum starved for 4 hrs and treated with 3% plasma for 24 hrs. Cells were initially fixed in 3% paraformaldehyde for 15 mins, prior to incubation in 0.2% Triton X-100 (Sigma-Aldrich) for 5 mins. Cells were blocked with 5% Bovine Serum Albumin (BSA) at room temperature for 30 mins prior to incubation with PGC-1α (1:200) (Novus Biologicals) overnight at 4 °C. Cells were then incubated with Alexa Fluor 488 goat anti-rabbit fluorescent secondary antibody (Invitrogen) at a 1:200 dilution. Cells were counterstained with 4,6-Diamidino-2-phenylindole dihydrochloride (DAPI) (10 μg/ml) to identify nuclei. Cells were visualized by fluorescent microscopy (Zeiss AxioImager M2). Mean fluorescent intensity was analyzed using Image J software in at least 10 random fields of view and compared to DMSO controls.

### Determination of mitochondrial mass

Mitochondrial mass was measured using MitoID Green fluorescent marker (Enzo Life Sciences) according to manufacturer’s instructions. MitoID Green is a cell-permeable small organic probe that spontaneously localizes to mitochondria regardless of their membrane potential. Briefly, cells were serum starved for 1hour and incubated with 3% plasma for 4hrs. Media was removed and cells were loaded with 500 μl 1X Assay buffer containing 0.5 μl MitoID Green Reagent and 0.5 μl Hoechst 33342 Nuclear Stain for 30 mins at 37 °C. Cells were then washed with PBS and fixed in 3% paraformaldehyde for 15 mins, prior to mounting. Mean fluorescent intensity was analyzed using Image J software in at least 10 random fields of view and compared to non-pregnant controls.

### Detection of mitochondrial superoxide by fluorescent microscopy

Intramitochondrial superoxide production was measured in treated HUVEC using the MitoSOX Red fluorescent reagent (Invitrogen). This fluorogenic dye selectively enters mitochondria within living cells where it is oxidized by superoxide anions. This oxidation reaction then emits a red fluorescence when bound to mitochondrial DNA. Cells were serum starved for 1hour and incubated with 3% plasma for 4hrs. Alternatively, cells were serum starved for 1hour, pre-treated with 5 μM Mito-Tempo, 5 uM N-acetylcysteine (NAC), or DMSO control for 2 hrs and incubated with 3% plasma for 4 hrs. Media was removed and cells were loaded with 0.5 uM MitoSox Red for 30 mins at 37 °C. Cells were then fixed and permeabilized prior to nuclear localization with DAPI. Mean fluorescent intensity was analyzed using Image J software in at least 10 random fields of view and compared to DMSO controls.

### Measurement of Mitochondrial O2 consumption

MitoXpress^®^ Xtra–Oxygen Consumption Assay (Luxel Biosciences) was used for the direct, real-time analysis of cellular respiration and mitochondrial function. MitoXpress^®^ Xtra is quenched by O_2_ through molecular collision, and thus the amount of fluorescence signal is inversely proportional to the amount of extracellular O_2_ in the sample. HUVEC were serum starved for 1 hour and incubated with 3% plasma for 4 hrs. After incubation cells were washed with clear respiration media. The MitoXpress-Xtra-HS probe was added to cells in accordance with manufacturer’s instructions. Oxygen consumption was measured using time-resolved fluorescence (TR-F) with a dual delay of 30 μs and 70 μs using a VarioSkan fluorescence plate reader (Thermo-Scientific). Rate of oxygen consumption was determined from the slope of fluorescence vs. time for each sample using relative fluorescence units/hour.

### Statistical Analysis

Data are shown as mean ± SEM, or fold change relative to vehicle control of at least 10 independent experiments. Mann Whitney U test or analyses of variance (ANOVA) were used where appropriate to determine statistical significance between groups in *in vitro* studies unless otherwise specified. Values of **P ≤ 0.01 and *P ≤ 0.05 were considered significant

## Additional Information

**How to cite this article**: McCarthy, C. and C. Kenny, L. Therapeutically targeting mitochondrial redox signalling alleviates endothelial dysfunction in preeclampsia. *Sci. Rep.* **6**, 32683; doi: 10.1038/srep32683 (2016).

## Supplementary Material

Supplementary Information
