# Supplementary material for: Therapeutically targeting mitochondrial redox signalling alleviates endothelial dysfunction in preeclampsia
Source: Sci Rep. 2016 Sep 8;6:32683. doi: 10.1038/srep32683 (PMC5015016; doi:10.1038/srep32683)
Supplement: Supplementary Information [file srep32683-s1.pdf]

THERAPEUTICALLY TARGETING MITOCHONDRIAL REDOX SIGNALLING ALLEVIATES ENDOTHELIAL DYSFUNCTION IN PREECLAMPSIA.

***Dr Cathal McCarthy, Senior Post-doctoral Scientist**

Professor Louise C Kenny, Professor of Obstetrics and Gynaecology

The Irish Centre for Fetal and Neonatal Translational Research (INFANT), Department of Obstetrics and Gynaecology, Cork University Hospital, Wilton, Cork, Ireland.

Supplementary Figure S1. Preeclampsia plasma mediated cell viability

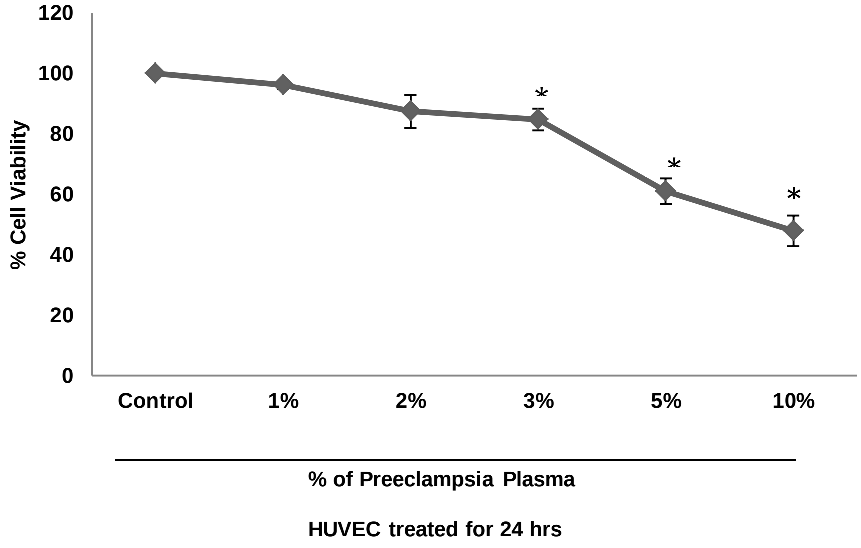

Dose dependent effect of preeclampsia plasma on HUVEC cell viability was assessed using a MTT assay. HUVEC were treated with varying percentages of preeclampsia plasma for 24hrs. Data are expressed as mean±SEM. (*P < 0.05 vs control). Data are representative of 3 independent experiments.

Supplemental Table S1. Characteristics of subjects from whom plasma samples were obtained

	**Uncomplicated Pregnancy**	**Pre-eclampsia**
**Maternal Age**	**30.67±1.08**	**29.17±0.93**
**BMI**	**24.08±0.79**	**25.89±0.96***
**Gestational Age**	**40.24±0.38**	**37.29±0.83***

The characteristics of subjects, 12 from each group of women are shown in the table. Data are shown as mean ± SEM. *denotes P<0.01 vs. uncomplicated pregnancy

Supplementary Table S2. Primer sequences used in real-time PCR

Primer Name	Sequence (5'-3')
SOD1 FP	AGC ATT AAA GGA CTG ACT GAA GG
SOD1 RP	GTC TCC AAC ATG CCT CTC TTC
SOD2 FP	GTT GGG GTT GGC TTG GTT TC
SOD2 RP	ATA AGG CCT GTT GTT CCT TGC
HO-1 FP	GGG TGA TAG AAG AGG CCA AGA CT
HO-1 RP	GCA GAA TCT TGC ACT TTG TTG CT
UCP-1 FP	GCT CCA GGT CCA AGG TGA AT
UCP-1 RP	ACA GCG GTG ATT GTT CCC AG
Endothelin-1 FP	TGC CAC CTG GAC ATC ATT TG
Endothelin-1 RP	GAC CTA GGG CTT CCA AGT CCA T
TNFα FP	CCC AGG GAC CTC TCT CTA ATC A
TNFα FP	AGC TGC CCC TCA GCT TGA G
hMitoF5	CTTCTGGCCACAGCACTTAAAC
hMitoR5	GCTGGTGTTAGGGTTCTTTGTTTT
hB2MF2	GCTGGGTAGCTCTAAACAATGTATTCA
hB2MR2	CCATGTACTAACAAATGTCTAAAATGGT
